# Hepatitis C virus nonstructural protein NS3 unfolds viral G-quadruplex RNA structures

**DOI:** 10.1016/j.jbc.2022.102486

**Published:** 2022-09-13

**Authors:** Binyam Belachew, Jun Gao, Alicia K. Byrd, Kevin D. Raney

**Affiliations:** Department of Biochemistry and Molecular Biology, University of Arkansas for Medical Sciences, Little Rock, Arkansas, USA

**Keywords:** Hepatitis C virus (HCV), NS3 helicase, G-quadruplex, G4 ligand, RNA helicase, RNA virus, enzyme kinetics, DEPC, diethyl pyrocarbonate, G4, G-quadruplex, G4RNA, G-quadruplex RNA, HCV, hepatitis C virus, NS3, nonstructural protein 3, PDS, Pyridostatin

## Abstract

Hepatitis C virus (HCV) is a major cause of liver-related diseases and hepatocellular carcinoma. The helicase domain of one of the nonstructural proteins of HCV, NS3 (nonstructural protein 3), is essential for viral replication; however, its specific biological role is still under investigation. Here, we set out to determine the interaction between a purified recombinant full length NS3 and synthetic guanine-rich substrates that represent the conserved G-quadruplex (G4)-forming sequences in the HCV-positive and HCV-negative strands. We performed fluorescence anisotropy binding, G4 reporter duplex unwinding, and G4RNA trapping assays to determine the binding and G4 unfolding activity of NS3. Our data suggest that NS3 can unfold the conserved G4 structures present within the genome and the negative strand of HCV. Additionally, we found the activity of NS3 on a G4RNA was reduced significantly in the presence of a G4 ligand. The ability of NS3 to unfold HCV G4RNA could imply a novel biological role of the viral helicase in replication.

Infection with Hepatitis C Virus (HCV) causes several liver-related health problems including hepatocellular carcinoma (1). It was estimated in 2015 that around 71.1 million people in the world were infected with the virus (2). In addition, a report from World Health Organization suggests that close to 290,000 people died in 2019 of liver issues linked to HCV infection https://www.who.int/news-room/fact-sheets/detail/hepatitis-c (Accessed November 8, 2021).

HCV is a positive-sense, ssRNA virus with a single ORF that encodes for a single polyprotein, which then is cleaved into structural and nonstructural proteins (1). The positive strand of HCV inside a host cell is copied to a negative strand RNA from which a large number of positive strands are made (1). The nonstructural proteins NS3, NS4A, NS4B, NS5A, and NS5B make up the complex that is necessary for viral replication (1, 3). Nonstructural protein 3 (NS3) is a dual-function protein with a protease domain at the N-terminus and a helicase domain at the C-terminus (1, 3). Studies suggest that the helicase activity of NS3 is essential for viral replication; however, its exact biological role in the life cycle of HCV is still under investigation (4, 5). The possible roles of NS3 helicase include the following: (1) unwinding the duplex formed by the positive and negative strand during replication, (2) unfolding secondary structures within the HCV genome, and (3) displacing proteins that could potentially deter the viral replication process (5, 6). NS3 is a member of the DExH helicase family, which is under superfamily 2 (7). It has unwinding activity on RNA or DNA substrates by translocating in the 3′ to 5′ direction (8).

In addition to unwinding dsRNA and DNA molecules, some helicases can resolve noncanonical DNA or/and RNA secondary structures known as G-quadruplexes (G4s) (9). G4 structures are formed when tandem repeats of guanine bases of an RNA or a DNA molecule interact with one another through Hoogsteen hydrogen bonding to form a planer structure known as a quartet or a tetrad (10). When two or more tetrads stack on top of each other, they form a stable G4 (9, 10, 11, 12, 13). G-quadruplex RNA (G4RNA) is more stable than its counterpart G4DNA (11). G4 stability is determined by the number of tetrads, loop length, and the number of strands involved in forming the tetrad (10, 11). G4 is also stabilized by monovalent cations such as K^+^ and Na^+^ that coordinate in the central channel of the four-stranded structure (11). Quadruplexes can further be stabilized by ligands such as Phen-DC3, NMM, TMPyP4, Pyridostatin (PDS) (9, 11).

In addition to the human genome, viral genomes could also harbor G4 structures (14). Recently, Lavezzo *et al*. explored the presence of potential G4-forming sequences in the genome of all human viruses discovered so far (14). The G4s in the genome of viruses could regulate translation, replication, and capsid formation (11).

Bioinformatics analysis on a number of HCV genotypes and subtypes revealed that both the positive and negative strands of the HCV genome contained conserved G4-forming sequences (15, 16). Folded G4s within viral genomes, particularly those stabilized further with ligands, could deter successful viral replication (15, 17, 18, 19). As a result, viruses need to control the folding and unfolding of these nucleic acid structures in a regulated manner. There are several helicases reported to have the ability to unfold RNA or/and DNA G4s (20, 21). For instance, helicases of the same family as NS3, such as DHX36 and DHX9, unfold G4RNAs (21, 22, 23, 24, 25). By unfolding these stable secondary structures, helicases remove the roadblocks to translation machineries (26, 27). HCV replication takes places at a vesicle termed the “membranous web (28)”. It is likely that NS3 is the only helicase inside the vesical because, according to the model proposed by Quinkert *et al.*, only molecules of a specific size (not bigger than 16 kDa) can enter through the “neck’ of the membranous web into the vesicle where replication takes place (29). Therefore, it is likely that HCV uses its own helicase (NS3) to unfold the conserved G4 structures within its genome to allow effective replication. In our study, we investigated the activity of NS3 on G4RNA-forming sequences derived from the positive or the negative strand of HCV. Our data suggests that NS3 unfolds G4RNA. However, its G4RNA-unfolding activity is significantly reduced when the G4 is inherently stable or stabilized with a G4 ligand.

## Results

### NS3 binds to HCV G4-forming substrates with or without a 3′ overhang

To determine whether and how NS3 interacts with G4RNA, we first design three different G-rich sequences: HCVG4, NEGG4, and NONHCVG4 (see Table S1 for sequences). HCVG4 contains the G-rich sequences from the positive strand of genotype 3a (15) that can theoretically form a three-tetrad G4 structure. NEGG4 substrate contains the conserved G-rich sequences from the negative strand of various strains of HCV (16) and has the potential to form a two-tetrad G4 structure. Unlike HCVG4 and NEGG4, NONHCVG4 does not represent a sequence from HCV; however, it is a useful model substrate because it can potentially fold into a highly stable three-tetrad G4 since it has only one nucleotide loop length. For G4RNA structures containing relatively shorter loops, loop length has an inverse relationship with G4 stability (30). Therefore, the shorter loop length results in more stable G4. NONHCVG4 was included in this study to understand how NS3 interacts with an RNA substrate that is likely to fold into an inherently stable G4.

The helicase domain of NS3 interacts with oligonucleotides in an orientation-specific manner (31, 32, 33). For instance, Levin and Patel reported that the HCV helicase bound to a duplex DNA substrate containing a 3′ overhang around 50 times tighter than a similar substrate with a 5′ overhang (33). In addition, Tai *et al*. and Gwack *et al*. independently demonstrated that effective duplex substrate–unwinding activity of NS3 helicase was dependent on the presence of a 3′ overhang (31, 32). Therefore, a 3′ overhang has a significant effect on the affinity and activity of NS3 on a partial duplex. With the aim of understanding the effect of a 3′ overhang on the interaction between NS3 and G4RNA, 20 nucleotide (nt) adenosine monophosphates (A_20_) were added to each of the above three substrates to form HCVG4-A_20_, NEGG4-A_20_, and NONHCVG4-A_20_.

**Figure 1:**
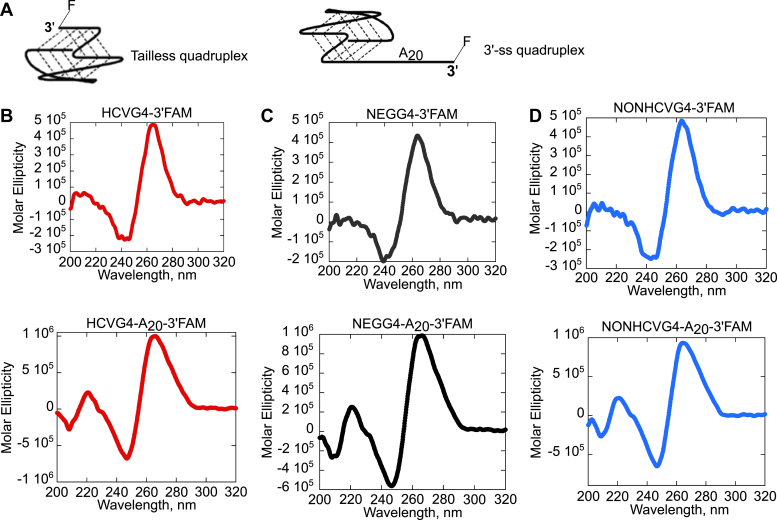
**CD spectra of G-rich RNA oligonucleotides with or without a 3′ overhang (tail) indicate the formation of parallel G4 structures in 100 mM KCl.***A*, diagram of an FAM-labeled G-quadruplex structure lacking (*left*) or containing (*right*) a 3′ overhang. Panels (*B*), (*C*), and (*D*) present the CD spectra of three different G-rich RNA oligonucleotides with (*bottom*) or without (*top*) a 3′ overhang. FAM, Fluorescein Amidite; G4, G-quadruplex.

CD spectra of all of the G-rich substrates mentioned above indicate that the sequences fold into a parallel G4 structures in 100 mM KCl (Fig. 1). A parallel G4 possesses a distinct CD spectrum with a peak around 265 nm and a valley around 240 nm (34, 35, 36). We conclude that the presence of 20-nt adenosine monophosphates at the 3′ end of HCVG4, NEGG4, or NONHCVG4 does not hinder the formation of a parallel G4.

**Figure 2:**
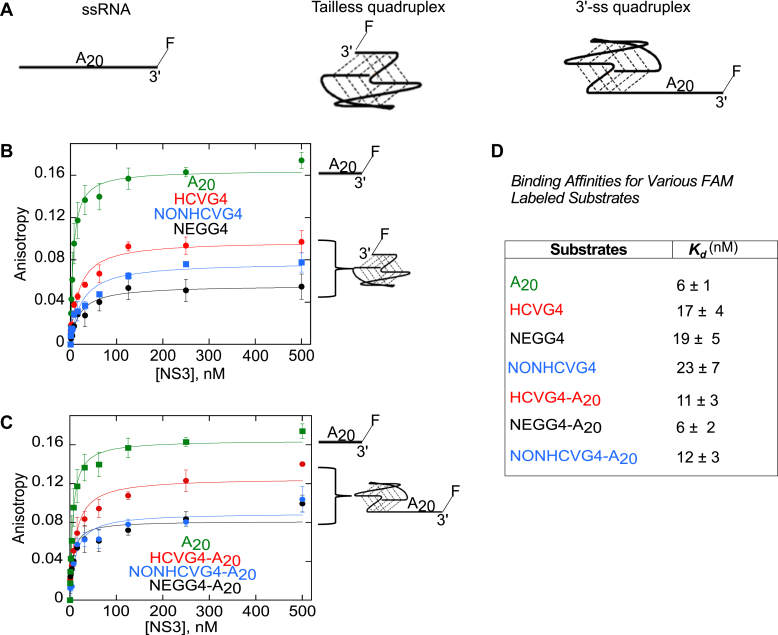
**NS3 binds tightly to G4-forming oligonucleotides with or without a 3′ overhang.***A*, the schematic representations of the three types of oligonucleotides, single-stranded RNA (ssRNA; positive control), tailless quadruplex, and 3′ tailed G4 structures. *B*, fluorescence anisotropy of increasing concentrations of NS3 with tailless G4s, HCVG4 (*red circles*), NONHCVG4 (*blue circles*), or NEGG4 (*black circles*). *C*, the fluorescence anisotropy measurements of NS3 with the 3′ tailed G4s, HCVG4-A_20_ (6 nM; *red circles*), NONHCVG4-A_20_ (6 nM; *blue circles*), NEGG4-A_20_ (5.4 nM; *black circles*). Anisotropy of the ssRNA is shown in *green* (*B* and *C*). The dissociation constants (*K*_*d*_) were determined by fitting the fluorescence anisotropy data to the quadratic equation, for each of the oligonucleotides listed in (*D*). The fluorescence anisotropy data presented in panels (*B*) and (*C*) represent the average of the values from at least three independent binding experiments. Error bars depict the SD. HCV, hepatitis C virus; G4, G-quadruplex.

To test whether NS3 binds to the G4RNA structures, a fluorescence anisotropy–binding assay was performed in 100 mM KCl binding buffer containing fluorescently labeled HCVG4, NEGG4, or NONHCVG4 and increasing concentration of NS3. NS3 bound tightly to the ssRNA, A_20_-3′FAM, with similar affinity (K_d_ = 6 ± 1 nM) to what was reported in the past for NS3 binding to a ssRNA (37) (Fig. 2). Because A_20_ has the highest amplitude and two NS3 molecules could potentially bind to the same oligonucleotide (based on eight nucleotides-binding site size for NS3 (38, 39)), we also fit the data points to a two-sites binding model and determined the K_d1_ and K_d2_ to be 4.9 nM and 860 nM, respectively (Fig. S1). NS3 bound to HCVG4, NEGG4, or NONHCVG4 tightly with K_d_ values in the nano-molar range (K_d_ = 17 ± 4 nM, 19 ± 5 nM, and 23 ± 7 nM, respectively) (Fig. 2, *A,* *B,* and *D*). Similarly, NS3 binds tightly to the 3′ overhang–containing substrates, HCVG4-A_20_, NEGG4-A_20_, or NONHCVG4-A_20_ (K_d_= 11 ± 3 nM, 6 ± 2 nM, and 12 ± 3 nM, respectively) (Fig. 2, *A,* *C,* and *D*). The binding affinity of NS3 to the 3′ overhang containing substrates is within two-fold of the single-stranded substrate. However, the substrates that lack a 3′ overhang bind to NS3 with a dissociation constant as high as four-fold than the ssRNA substrate. Nonetheless, the absence of a single-strand protein-loading zone does not prevent the NS3 from binding to the G4-forming substrates.

### NS3 is active in a buffer that supports G4RNA folding

**Figure 3:**
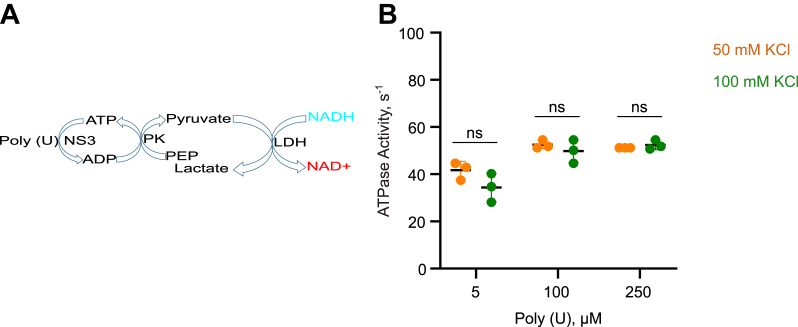
**NS3 is active in a buffer that supports G4 folding.***A*, to test the effect of 100 mM KCl, the salt concentration at which G4 unfolding was studied, on the activity of the purified NS3, we performed an enzyme-coupled spectrophotometric ATPase assay which couples NADH oxidation with ATP hydrolysis. *B*, the assay was carried out in a reaction buffer containing 50 mM KCl (*B*; *orange*) or 100 mM KCl (*B*; *green*). ATP hydrolysis by NS3 (50 nM) was initiated with 5 μM, 100 μM, or 250 μM Poly (U). Experiments were performed in triplicate. Error bars depict SD. The difference between two groups is not significant (ns) when *p* > 0.05. *p* values were calculated from independent-samples t-tests. G4, G-quadruplex.

Unfolding of a G4 structure requires a reaction buffer containing enough cations to maintain the folding of the G4 during the span of the reaction. Therefore, it is necessary to first verify the activity of the helicase in a buffer in which the G-rich oligonucleotides fold to form a stable G4. Several previous studies that examined the interaction of a helicase with a G4-forming oligonucleotides reported of using a buffer containing 100 mM KCl (26, 40, 41). Therefore, we first determined the ATPase activity of a recombinant NS3 protein in 100 mM KCl buffer by conducting enzyme-coupled spectrophotometric ATPase assay that links ATP hydrolysis with the oxidation of NADH (Fig. 3*A*). In saturating conditions, which is the concentration of Poly (U) at which the specific activity is maximum, NS3 has ATPase activity of 52.5 ± 1.8 s^−1^ in low salt (50 mM KCl) buffer and 49.8 ± 5.0 s^−1^ in high salt (100 mM KCl) buffer, which is not significantly different (Fig. 3*B*). Therefore, the purified NS3 is as active at high salt concentration as it is at low salt concentration.

### The ability of NS3 to unfold G4 could be inferred from enzyme-catalyzed G4 reporter duplex unwinding assay

**Figure 4:**
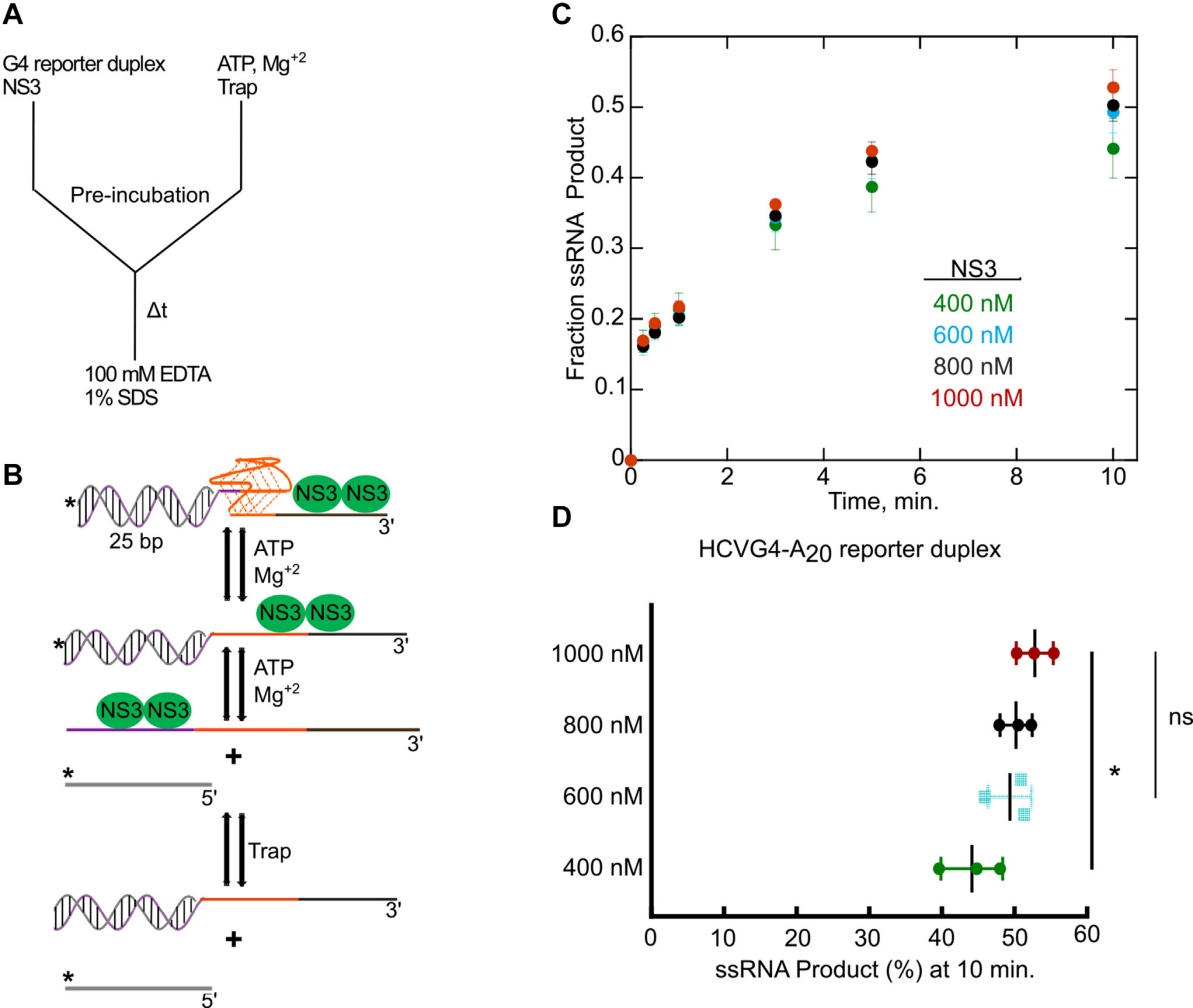
**Determining saturating enzyme concentration for the G4 reporter duplex unwinding reaction.** The reaction setup for the G4 reporter duplex unwinding reaction is shown in (*A*). The enzyme was preincubated with the G4 reporter substrate before addition of ATP. The reaction was quenched at increasing time points in a buffer containing 100 mM EDTA and 1% SDS. In this reaction, the unfolding of the G4 structure precedes the unwinding of the duplex region (*B*). A trapping strand is added to deter the reannealing of the unwound substrate (*B*). ∗ symbol in (*B*) indicates that the substrate is radiolabeled. *C*, in an effort to define the enzyme concentration that saturates the G4 reporter duplex substrate, we measured the amount of product formed with increasing concentrations of NS3 under multi-turnover conditions. The product formed with 400 nM, 600 nM, 800 nM, and 1000 nM NS3 concentrations are represented in *green*, *blue* (*C*) or *cyan squares* (*D*), *black*, and *red colors*, respectively (*C* and *D*). The amount of product measured at 10 min for each NS3 concentrations is presented in (*D*). Experiments were performed in triplicate. Error bars depict SD. The difference between two groups is statically significant when *p* ≤ 0.05 (∗) and not significant (ns) when *p* > 0.05. *p* values were determined from a one-way ANOVA tests for three or four independent groups. G4, G-quadruplex.

To test whether NS3 can unfold G4-forming substrates to which it binds, we conducted a G4 reporter duplex unwinding assay. A radiolabeled–G4 reporter duplex substrate that contains the G-rich sequences from HCVG4, NEGG4, or NONHCVG4 oligonucleotides flanked by a 25 base pair duplex region and A_20_ 3′ overhang was made as described in the Experimental procedures and illustrated in Fig. S2. Three nucleotides were added as a linker between the duplex region and the G4-forming sequences to allow proper folding of the G4 structure. The reporter substrate was incubated with the enzyme and the reaction was initiated with the addition of ATP (Fig. 4, *A* and *B*). In the reporter assay, the unfolding of the G4 structure by the enzyme is reported by the unwinding of the duplex region (Fig. 4*B*).

Initially, we evaluated the amount of NS3 needed for the G4 reporter assay to saturate the substrate. We carried out the G4 reporter assay with varying concentrations of NS3 (400 nM, 600 nM, 800 nM, or 1000 nM) and 2 nM HCVG4-A_20_ reporter duplex substrate. The product formed from 400 nM NS3 was significantly lower than the product formed from the other NS3 concentrations; however, the amount of products obtained from 600 nM, 800 nM, and 1000 nM NS3 are not significantly different (Figs. 4, *C*, *D* and S3), suggesting 600 nM NS3 is enough to saturate the substrate. Consequently, 600 nM NS3 is the concentration used for subsequent dsRNA or G4 reporter duplex unwinding reactions.

### NS3 unfolding of G4 RNA is affected by substrate sequence and reaction conditions

**Figure 5:**
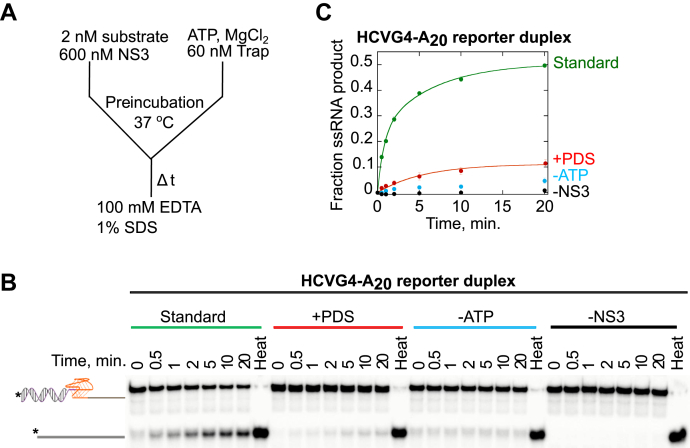
**NS3 unfolds the G4 structure within HCVG4-A**_**20**_**reporter duplex substrate; however, its activity is significantly reduced in the presence of a G4-stabilizing ligand.***A*, the standard reaction condition (*green*) involved the following factors: 2 nM HCVG4-A_20_ reporter duplex, 600 nM NS3, and 5 mM ATP. For the + PDS (*red*), -ATP (*blue*), and –NS3 (*black*) unwinding reactions, the same factors as the standard reaction were involved except that 0.25 μM PDS was added, or 5 mM ATP was left out, or the reaction was carried out in the absence of NS3, respectively (*B* and *C*). The ratio of product to substrate for each reaction time is plotted (*C*). The data for the standard reaction were fit to a double exponential equation with rate constant values of *k*_*1*_ =1.29 ± 0.28 min^−1^; *k*_*2*_ = 0.16 ± 0.04 min^−1^. Data from the experiment including PDS were best fit to a single exponential equation with a rate constant value of *k* = 0.18 ± 0.03 min^−1^. Experiments were executed in triplicate. Error bars depict SD. HCV, hepatitis C virus; G4, G-quadruplex; PDS, Pyridostatin.

We used these reaction conditions as our standard condition: 600 nM NS3 incubated with 2 nM-radiolabeled HCVG4-A_20_ reporter duplex substrate in a buffer containing 100 mM KCl (Fig. 5*A*). Under standard conditions, NS3 unwinds HCVG4-A_20_ reporter duplex with an amplitude of product formation of 50 ± 1% at 20 min. The product formation is drastically reduced to 11 ± 1% when 0.25 μM PDS, a known G4-stabilizing ligand, is added to the reaction (Fig. 5, *B* and *C*). The same concentration of PDS has less effect on the activity of NS3 on a partial duplex substrate with the same length and sequence as the HCVG4-A_20_ reporter duplex, except that the guanines within the G-rich sequence are mutated to adenine (MUTHCVG4-A_20_; Table S1 and Fig. S4). For the HCVG4-A_20_ reporter duplex unwinding, there is a small (<5%) but measurable amount of product observed in the absence of ATP during the course of the reaction. In contrast, virtually no product is measured in the absence of NS3, suggesting that the product observed under the standard reaction conditions is driven by the enzyme. Interestingly, some product is measured from the zero second-time point (Fig. 5*B*). It is possible that this product is formed during preincubation in an ATP-independent manner. Reynolds *et al*. reported NS3 unwinding of 3′- or 5′-tailed short duplexes in an ATP-independent manner and proposed a noncanonical duplex melting mechanism by NS3, which involved local strand separation utilizing binding energy (42). Therefore, the product we observe from the G4 reporter duplex unwinding reaction in the absence of ATP could be due to NS3 binding and unwinding of the duplex region adjacent to the G4 structure in an ATP-independent manner.

**Figure 6:**
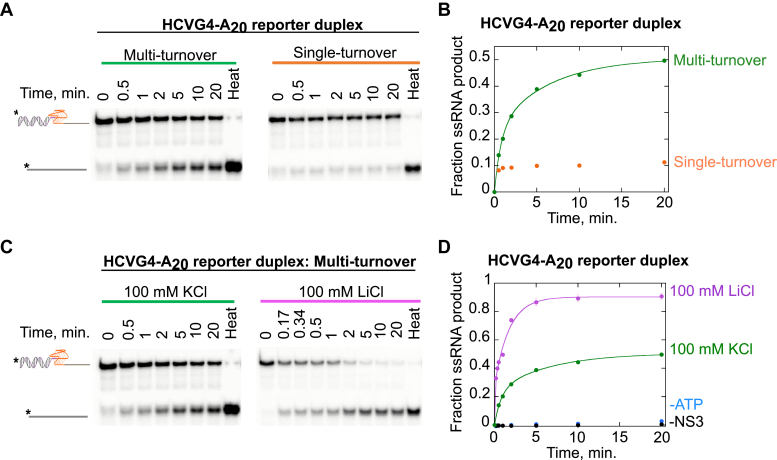
**NS3 unfolding of HCVG4 is affected by reaction conditions.***A*, the substrate and product from NS3 unfolding HCVG4 then unwinding the reporter duplex under multi-turnover or single-turnover conditions in 100 mM KCl are separated by PAGE. The gel image was quantified and the ratio of product to substrate for a given time point was plotted against time (*B*), with *green* and *orange circles* representing the data points for multi-turnover and single-turnover conditions, respectively. The gel image of HCVG4-A_20_ reporter duplex unwinding reaction in 100 mM KCl or LiCl is shown in (*C*), and the quantification of the gel image is presented in graph (*D*), with *green* and *pink circles*, respectively. Conditions in the absence of ATP (*blue circles*) or NS3 (*black circles*) were performed under the same conditions as the 100 mM LiCl reaction except that ATP or NS3 was omitted from the reaction (*D*). The data for multi-turnover (*A* and *B*) and 100 mM KCl (*C* and *D*) are re-represented from Figure 5 for comparison. The gel images for multi-turnover (*A*) and 100 mM KCl (*C*) are the same as the gel image shown in Figure 5*B* for the standard reaction. Experiments were performed in triplicate. Error bars represent the SD. HCV, hepatitis C virus; G4, G-quadruplex.

A single-turnover reaction involves product formation by the actions of NS3 molecules that are bound to the substrate at the initiation of the reaction. To create single-turnover reaction conditions, a protein trap is added in the reaction buffer to keep the dissociated NS3 molecules from rebinding to the substrate. The amount of product formed from a single-turnover reaction indicates the level of processivity of NS3 with the substrate studied. Under single-turnover conditions, 11 ± 1% product is formed from HCVG4-A_20_ reporter duplex, which is a 78% reduction from the product measured under a multi-turnover reaction condition in which NS3 molecules are allowed to bind, unwind, dissociate, and rebind for another cycle of enzymatic action (Fig. 6, *A* and *B*). The low product formation from the single-turnover reaction indicates NS3 might need to undergo a series of association and dissociation events from the substrate in order to unfold the G4 and then unwind the adjacent duplex region.

Unlike KCl and NaCl, LiCl does not support the formation of a stable G4 (43). To test the effect of not having a stable G4 on the unfolding activity of NS3, we allowed the enzyme to unfold the same substrate, HCVG4-A_20_ reporter duplex, in 100 mM LiCl reaction buffer, which resulted in 91 ± 2% product formation (Fig. 6, *C* and *D*). The product obtained in 100 mM KCl is significantly lower than in 100 mM LiCl, implying that a stable G4 could be a formidable barrier, keeping the NS3 from unwinding the adjoining duplex region of the G4 reporter duplex substrate.

**Figure 7:**
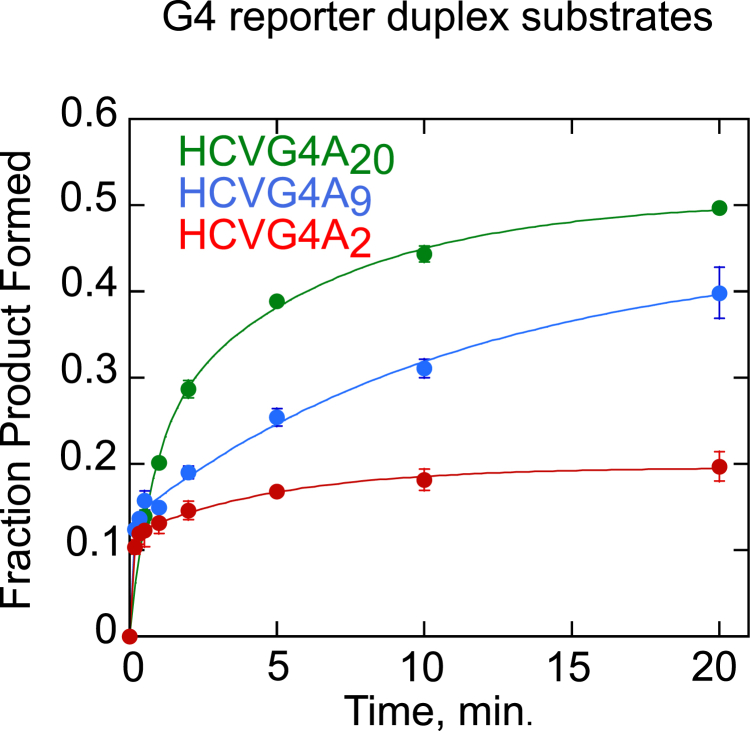
**HCVG4 unfolding activity of NS3 is directly proportional to the length of the 3′ overhang.** NS3-dependent unwinding of HCVG4 reporter duplex substrates with 20, 9, or 2 nucleotides of single-stranded RNA on the 3′ overhang was carried out, and the fraction product formed was calculated and presented with *green*, *blue*, and *red circles*, respectively. The data for HCVG4-A_20_ are replotted from Figure 5 for comparison. The experiments were conducted in triplicate, and error bars depict SD. HCV, hepatitis C virus; G4, G-quadruplex.

In addition to the external factors discussed above, the length of the 3′ overhang could also regulate the G4-unfolding activity of NS3. Efficient partial duplex unwinding by NS3 is highly dependent on the length of the 3′ overhang; the longer the tail, the higher the unwinding amplitude (44). In order to investigate the effect of the length of a 3′ overhang on G4-unfolding activity of NS3, a G4 reporter duplex unwinding reaction was conducted with HCVG4 reporter substrate containing 20-nt, 9-nt, or 2-nt 3′ overhangs. We observed 50 ± 1%, 40 ± 3%, and 20 ± 2% product at 20 min for substrates with 20-nt, 9-nt, and 2-nt of 3′ overhangs, respectively, in a multi-turnover reaction (Fig. 7). The result strongly implies that the length of the single-strand extension is important for efficient unfolding of a G4 by NS3.

**Figure 8:**
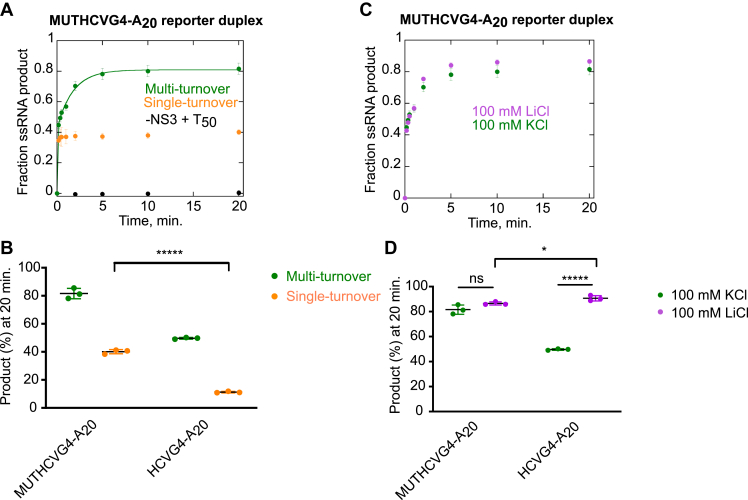
**G-quadruplex is a strong deterrent against the helicase activity of NS3.***A*, the G4-forming region within the HCVG4-A_20_ reporter duplex was mutated to create a control duplex (MUTHCVG4-A_20_) of the same length but unable to fold into a G4 structure. The amount of product formed at increasing time points from the control substrate in the presence of NS3 under multi-turnover (*green*) or single-turnover (*orange*) conditions and a reaction that contained T_50_ protein trap but no enzyme (*black*) are shown. *B*, under multi-turnover (*green*) or single cycle (*orange*) conditions, NS3 had significantly higher activity on MUTHCVG4-A_20_ than on HCVG4-A_20_. *C*, no significant difference in product formation was observed when NS3 unwound MUTHCVG4-A_20_ in a reaction buffer containing KCl (*green*) or LiCl (*pink*). *D*, in contrast, the product obtained from HCVG4-A_20_ was significantly lower in 100 mM KCl (*green*) than in 100 mM LiCl (*pink*). Each of the reactions were conducted under multi-turnover conditions unless otherwise specified. The data for HCVG4-A_20_ in (*B* and *D*) are replotted from Figure 6 for comparison. Each of the reactions were performed in triplicate. Error bars depict SD. The difference between two groups is statistically significant when *p* ≤ 0.05 (∗), *p* ≤ 0.00001 (∗∗∗∗∗). *p* > 0.05 implies that the difference between two groups is not statistically significant (ns). *p* values were calculated from independent-samples t-tests. HCV, hepatitis C virus; G4, G-quadruplex.

Under multi-turnover conditions, 82 ± 4% of the MUTHCVG4-A_20_ control duplex is unwound, which is significantly higher than the product obtained from HCVG4-A_20_ reporter duplex substrate (50 ± 1%) (Fig. 8, *A* and *B*). In addition, the fraction of the duplex substrate unwound under single-turnover conditions was considerably higher than the amount of G4 containing reporter duplex unwound (Fig. 8, *A* and *B*). This higher activity of NS3 with the control duplex substrate than with the G4 reporter duplex implies that the helicase is more processive on the former than on the later. It also indicates that G4 is a strong deterrent for helicase progression. Unlike for HCVG4-A_20_ reporter duplex unwinding, the type of cation in which the unwinding reaction takes place does not make a significant difference for MUTHCVG4-A_20_, which suggests that at the concentration tested, the KCl does not interfere with the helicase activity of NS3 (Fig. 8, *C* and *D*). Therefore, the lower level of product formed from the G4-containing reporter duplex in 100 mM KCl (Fig. 8, *B* and *D*) is due to the presence of a G4.

We also deleted the G-rich sequence within the HCVG4-A_20_ reporter substrate to create another control substrate containing 25-mer duplex and 20-mer 3′ overhang (DELHCVG4-A_20_; Table S1). The unwinding of DELHCVG4-A_20_ resulted in more product formation compared to the WT substrate (Fig. S5), supporting the conclusion that the G-rich sequence within HCVG4-A_20_ reporter duplex is responsible for reducing the unfolding activity of NS3 in 100 mM KCl.

**Figure 9:**
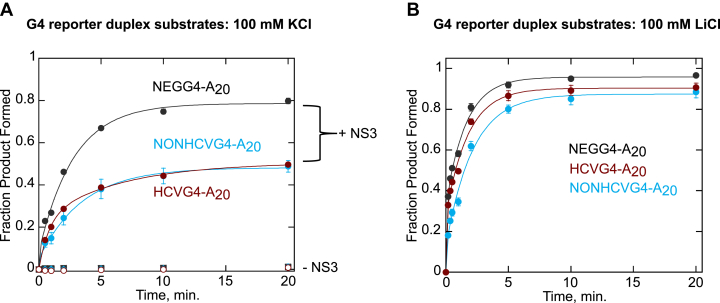
**Both oligonucleotide sequence and the type of monovalent cation present in the reaction buffer affect NS3 G4RNA unfolding.** Data from NS3-catalyzed unwinding of three G4 substrates with a reporter duplex (NEGG4-A_20_, NONHCVG4-A_20_, and HCVG4-A_20_ reporter duplexes) are presented. The reactions were carried out in a buffer containing 100 mM KCl (*A*) or LiCl (*B*). Data points for the reactions containing NS3 are depicted on graphs with *black*, *blue*, or *red solid circles* representing NEGG4-A_20_, NONHCVG4-A_20_, or HCVG4-A_20_, respectively (*A* and *B*). Unwinding reactions in the absence of NS3 are shown in *black solid square*, *blue solid diamond, or red open circles* representing NEGG4-A_20_, NONHCVG4-A_20_, or HCVG4-A_20_, respectively (*A*). The data for HCVG4-A_20_ (100 mM KCl) are replotted from Figure 5 for comparison. The data for HCVG4-A_20_ (100 mM LiCl) are replotted from Figure 6 for comparison. Experiments were performed in triplicate and error bars represent SD. HCV, hepatitis C virus; G4, G-quadruplex; G4RNA, G-quadruplex RNA.

To test the effect of oligonucleotide sequence on the ability of NS3 to disrupt G4, we conducted G4 reporter duplex unwinding assays with three different RNA G4-forming reporter substrates: HCVG4-A_20_, NEGG4-A_20_, or NONHCVG4-A_20_. NS3-catalyzed HCVG4-A_20_, NEGG4-A_20_, and NONHCVG4-A_20_ reporter duplex unwinding reactions in 100 mM KCl resulted in 50 ± 1%, 80 ± 1%, 49 ± 3% product formation, respectively (Fig. 9*A*). The activity of NS3 on the three-tetrad G4-forming substrates (HCVG4-A_20_ and NONHCVG4-A_20_) is significantly lower than on a two-tetrad G4-forming substrate (NEGG4-A_20_). No significant difference in product formation among the three substrates was observed when the unwinding reaction contained 100 mM LiCl (Fig. 9*B*), further confirming the conclusion that NS3 unfolding is affected by G4 stability, which in turn is determined by oligonucleotide sequence and the type of monovalent cation used.

The gel images from the unwinding reactions of the above three reporter substrates (Fig. S6) indicate that NONHCVG4-A_20_ forms a significant amount of higher order structure (intermolecular G4) in 100 mM KCl, but not in 100 mM LiCl buffer (Fig. S6, *E* and *F*). NEGG4-A_20_ also forms intermolecular G4 in 100 mM KCl (Fig. S6, *C* and *D*). Therefore, when analyzing the fraction G4 unfolded from NONHCVG40-A_20_- or NEGG4-A_20_- unwinding reaction (Fig. 9), we took the intermolecular G4s present at each time points into consideration when quantifying the results. On the other hand, since the amount of intermolecular G4 observed is negligible (Fig. S6,*A* and *B*), the analysis of the fraction of G4 unfolded from HCVG4-A_20_ reporter substrate did not involve intermolecular G4 structures.

### Examination of NS3 unfolding of G4RNA by G4-trapping assay

**Figure 10:**
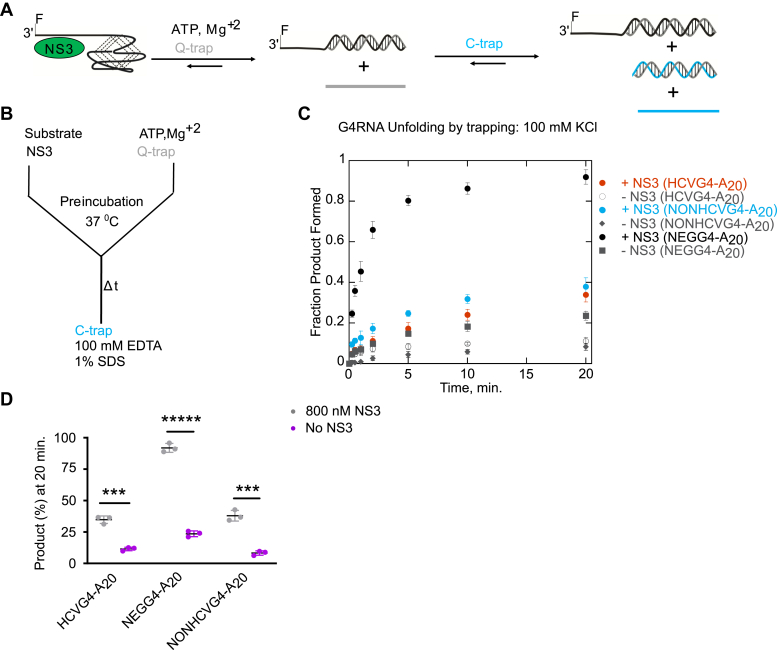
**Trapping assay allows direct measurement of G4RNA unfolding by NS3.** A schematic representation of NS3-catalyzed G4RNA unfolding reaction using a trapping strand is shown in (*A*) and the reaction setup is presented in (*B*). The ability of NS3 to unfold 25 nM of FAM-labeled (indicated by F) HCVG4-A_20_, NEGG4-A_20_, or NONHCVG4-A_20_ was assessed. NS3 was incubated with the substrates. Upon addition of ATP and Mg^+2^, the unfolded G4 sequences were trapped by the Q-trap. The reaction was quenched in a buffer containing EDTA, SDS, and C-trap. The C-trap formed a duplex with excess Q-trap to prevent additional unfolding of the substrate after the reaction ended. *C*, the product formed from NEGG4-A_20_, NONHCVG4-A_20_, and HCVG4-A_20_ unfolding in the presence of NS3 is shown in *solid black*, *blue*, and *red circles*, respectively. For the reaction with trap alone (no NS3), the products formed from NEGG4-A_20_, NONHCVG4-A_20_, and HCVG4-A_20_ are shown in *grayopen circles*, *diamonds*, and *squares*, respectively. *D*, the product formed at 20 min from each of the substrates with (*gray*) or without (*pink*) NS3 is shown. The data for HCVG4-A_20_ in the presence of NS3 (*C* and *D*) are replotted from Fig. S7 for comparison. The experiments were performed at least in triplicate. Error bars signify SD. The difference between two groups is statically significant when *p* ≤ 0.001 (∗∗∗) or *p* ≤ 0.00001 (∗∗∗∗∗). *p* values were calculated from independent-samples t-tests. HCV, hepatitis C virus; G4, G-quadruplex; G4RNA, G-quadruplex RNA; FAM, Fluorescein Amidite.

**Figure 11:**
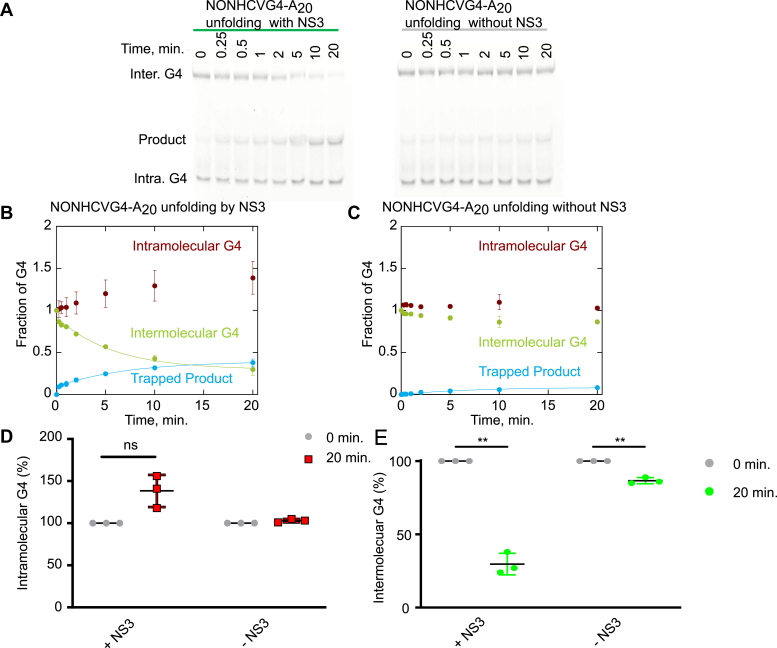
**G4RNA-unfolding activity of NS3 is completely inhibited by intramolecular G-quadruplex.***A*, FAM-labeled NONHCVG4-A_20_ formed both intramolecular G4 and intermolecular G4 (higher order structure) in the presence of 100 mM KCl. The native polyacrylamide gel image shown in (*A*) displays the product formed from a trapping assay conducted in the presence (*left*) or absence (*right*) of NS3. The quantity of intramolecular or intermolecular G4 in the presence (*B*) or absence (*C*) of NS3 over time is presented in line graph. The trapped product data shown in panel (*B*) or (*C*) are replotted from Figure 10 for comparison. The data points for the trapped product in (B) are fit to a double exponential equation and the *k*_1_= 9.5 ± 2.8 min^−1^ and *k*_2_= 0.14 ± 0.0098 min^−1^. The intermolecular G4 data points (*B*) are fit to a single exponential decay and the *k* = 0.17 ± 0.044 min^−1^. The data points for the trapped product in the absence of NS3 (*C*) are fit to a single exponential equation and *k* = 0.14 ± 0.020 min^−1^. The scatter plots in (*D* and *E*) exhibits the amount of intramolecular (*D*) or intermolecular (*E*) G4 unfolded in the presence or absence of NS3 at 0 (*gray circles* for both panel *D* and *E*)or 20 min (*red squares* for panel *D* and *green circles* for panel *E*). The experiments were performed in triplicate. Error bars signify SD. The difference between two groups is statistically significant when the *p* value ≤ 0.01 (∗∗) and not significant (ns) when *p* value is > 0.05. *p* values were calculated from dependent-samples t-tests. FAM, Fluorescein Amidite; G4, G-quadruplex; G4RNA, G-quadruplex RNA; HCV, hepatitis C virus.

Because of the G4 reporter duplex substrate consists of a duplex region linked to a G4, unfolding of the G4 precedes the unwinding of the duplex. Consequently, the overall rate of the G4 reporter unwinding reaction is the sum of the rates of G4 unfolding and duplex unwinding. There is a possibility that G4 reporter assay could overestimate the actual G4 unfolding in situations where the duplex region is unwound by direct binding of NS3 without translocating along the G-rich sequences. It could also underestimate the amount of G4 unfolded in cases where the duplex unwinding is much slower than the G4 unfolding. To overcome such scenarios, we decided to further investigate NS3 G4RNA unfolding by a G4-trapping method where NS3 interacts with a G4-containing substrate and the unfolded product is captured by a complementary strand (Q-trap) (Fig. 10, *A* and *B*). The duplex product and the G4 substrate can then be separated by gel electrophoresis due to the difference in their migration speed (Fig. 11*A*). A second trap, C-trap, is present in the quench buffer to prevent the Q-trap from unfolding the substrate. We conducted the G4-trapping assay at saturating conditions (Fig. S7) with respect to the substrate. The amplitude of product formation in the presence of NS3 from HCVG4-A_20_, NEGG4-A_20_, and NONHCVG4-A_20_ are 35 ± 3%, 92 ± 4%, and 38 ± 4%, respectively (Fig. 10, *C* and *D*). The products formed by the Q-trap alone, on the other hand, are 12 ± 1%, 24 ± 2%, and 8 ± 2%, respectively (Fig. 10, *C* and *D*). For each substrate, NS3-dependent unfolding is drastically higher than unfolding by Q-trap, which indicates that the complementary strand alone has only a minimal effect on G4RNA unfolding. Similar to the G4 reporter–unwinding reactions, the most product is formed from HCVNEGG4-A_20_ unfolding, most likely due to its lower stability. However, there is no significant difference between the products obtained from NONHCVG4-A_20_ and HCVG4-A_20_. The gel image for NONHCVG4-A_20_–unfolding experiment shows three major bands (Fig. 11*A*). The bands represent product, intramolecular G4 species (migrates faster than the product), and intermolecular G4 species or higher order G4 structure (migrates slower than the product). Interestingly, the NS3 is completely inactive on the intramolecular G4 subpopulation (Fig. 11, *B* and *D*). However, the intermolecular G4 is disrupted by NS3 more so than by the complementary strand (Fig. 11, *B*, *C*, and *E*). Therefore, the 38 ± 4% product formed from NONHCVG4-A_20_ is due to the disruption of the intermolecular G4 species not the intramolecular G4 species, which are too stable to be unfolded by NS3 or the complementary strand.

## Discussion

The binding experiment shows that NS3 binds with high affinity to G4-containing substrates with or without a 3′ overhang (Fig. 2). Zhou *et al*. reported that the affinity of NS3 full length from genotype 1b for RNA duplex was drastically reduced with shorter 3′ overhang length (44), signifying the need for having a 3′ overhang for NS3 to efficiently bind to a partial duplex. Similarly, NS3 binds with higher affinity to G4-containing substrates with a 3′ overhang than without (Fig. 2), which suggests the importance of a 3′ overhang for NS3-dependent unfolding of G4 structures. Unlike certain RNA helicases, NS3 does not show preferential binding to G4RNA compared to ssRNA. By performing EMSAs, Liu *et al*. demonstrated DHX36’s ability to bind to RNA G4 of C9orf72 repeat with higher affinity than substrates that form antiparallel G4, hairpins, or non-G4–forming sequences (26). Gao *et al*. also reported that the DEAD box helicase Mss116 bound four-fold tighter to a yeast two-tetrad quadruplex than an ssRNA (45). The preference of NS3 for ssRNA to G4 structure–containing RNA substrate could be an indication of the potential mechanism it possesses to effectively unfold a G4 structure. NS3 might first bind to the single-stranded region and form a functional complex with the substrate. Then, it could translocate in the 3′ to 5′ direction toward the G4 structure using the energy from ATP hydrolysis. Other helicases are also thought to unfold G4 structures by translocation-based mechanism (9, 41).

The change in fluorescence anisotropy of the ssRNA is greater than that of the substrates with G4-forming sequences (Fig. 2). The minimum binding site for NS3 is eight nucleotides (38, 39); therefore, it is likely that more than one molecule of NS3 is binding to the same ssRNA. The fact that the change in anisotropy for substrates lacking a 3′ overhang is smaller could be an indicative of the binding of fewer NS3 molecules than the ssRNA. It was surprising to us that the change in anisotropy of the G4-containing substrates with a 3′ overhang is significantly smaller than that measured for the ssRNA (Fig. 2). It is possible that the presence of a G4 structure in these substrates could affect the single strand extension, reducing the size of the loading site and allowing the binding of fewer NS3 molecules.

Our data suggest that efficient unfolding of G4RNA by NS3 depends on factors such as reaction conditions, substrate sequence, and length of the 3′ overhang adjacent to the G4 structure. NS3 exhibits low activity on G4-containing substrates under conditions that support stable G4 formation (Figs. 5 and 6). G-rich substrates that fold into a two-tetrad rather than a three-tetrad G4 structure are better substrates for NS3 (Fig. 9). Our results strongly suggest that even if NS3 can unfold G4RNA, doing so requires multiple rounds of ATP hydrolysis over a relatively long period of time when the G4 is further stabilized by small molecules (Fig. 5) or encounters inherently stable intramolecular G4 (Fig. 11). The virus could overcome this low G4-unfolding activity of NS3 by engaging other viral proteins in the process. It is possible that accessory proteins could assist NS3 with G4 unfolding. Rajagopal and Patel reported single-stranded–binding proteins could enhance the processivity of the helicase domain of NS3 on a DNA substrate (46). In addition, Zhang *et al* demonstrated that NS5B could stimulate the helicase activity of NS3 by seven-fold (47). Similarly, G4 unfolding by NS3 could be affected by NS5A or/and NS5B. In addition, our data indicate that longer 3′ extensions enhance the efficiency of G4RNA unfolding by NS3 (Fig. 7). The 3′ overhang could allow the productive assembly of multiple molecules of NS3, which could lead to cooperative unfolding of a G4. This proportional relationship between enzyme activity and the length of a 3′ overhang is also observed with other helicases. For example, partial duplex DNA-unwinding efficiency of the helicase domain of NS3 (NS3h) (48), bacteriophage T4 Dda (49), and pif1 (50) helicases increased as a function of the length of the ssDNA tail.

In addition to HCV, other positive-sense ssRNA viruses such as Zika, MERS-CoV, SARS-CoV, and SARS-CoV-2 also contain G4-forming sequences (51, 52). In fact, potential G4-forming sequences are reported to exist in many other viral genomes (14). One possible evolutionary advantage of viral G4 structures is to allow the virus to avoid the host immune detection by maintaining viral protein production at a level that is sufficient for persistent infection but below the threshold for initiation of the host immune response (53). If G4 structures remain folded, they can potentially block translation and replication machineries (15). No matter their function, viruses need to regulate the folding and unfolding of G4 structures in a controlled manner. There are several reasons to believe that NS3 could unfold HCV G4 structures *in vivo*. One reason could be its high affinity for G4-containing substrates (K_d_ values in the low nM range; Fig. 2). Second, there are excess copies of NS3 for every positive or negative strand RNA present within an active HCV replicase complex (29). Having an excess of NS3 within the replication complex increases the likelihood of NS3 interacting with HCV G4 structures. Moreover, it is likely that NS3 is the only helicase protein in the membranous web (site of replication) which keeps large molecules from entering (>16 KDa) into the vesicle (29). G4RA unfolding by NS3 could be a novel role for the viral helicase in the life cycle of HCV.

There have been attempts to target viral G4s and suppress replication. For example, the HIV *nef* gene contains conserved G4 structures which, when stabilized by TMPyP4 (a G4 stabilizer), reduces nef-GFP protein expression in a GFP-based reporter gene assay and nef-dependent HIV-1 infectivity (54). Wang *et al*. found treating HCV-infected Huh-7.5.1 cells with G4-stabilizing ligands results in reduced viral RNA and protein levels (15). In the present study, the addition of PDS significantly reduced the ability of NS3 to unfold HCV G4 (Fig. 5), suggesting the potential role of G4-stabilizing ligands as anti-HCV therapeutic agents. There have been many attempts to develop NS3 helicase inhibitors as anti-HCV therapeutic agents (55). However, not a single NS3 helicase inhibitor is made available for a market use due to toxicity that stems from off target effects (55). We suggest that a combination drug in the form of NS3 inhibitor and HCV G4 stabilizer might be more effective in reducing viral replication as opposed to inhibition of a single target.

## Experimental procedures

### Oligonucleotides and protein

The RNA and DNA oligonucleotides used in this study were purchased from Dharmacon and Integrated DNA Technologies, respectively. Table S1 displays the sequences of RNA oligonucleotides. The oligonucleotides were purified as explained in (40), but with minor modifications. RNA oligonucleotides were gel purified, electroeluted, desalted, and then dried by vacuum centrifugation. The dried oligonucleotides were deprotected as directed by the manufacturer before resuspension in a sodium-free storage buffer containing 10 mM Hepes, pH 7.5 and 1 mM EDTA, pH 8.0. All buffers or solutions in this study were made in diethyl pyrocarbonate (DEPC)- (ACROS Organics) treated water to make them RNase free. 0.1% DEPC in deionized water was prepared and then autoclaved before use. The concentrations of the purified oligonucleotides were determined using calculated extinction coefficients or the extinction coefficient provided by the manufacturer. For the G4 reporter assay, the trap reporter substrate was radiolabeled as reported in (56) with a few changes. Here, the RNA substrate was incubated with [γ -^32^P] ATP (PerkinElmer) in the presence of T4 polynucleotide kinase (Promega) for 1 h at 37 °C. Then, unlabeled excess [γ -^32^P] ATP was removed using Illustra MicroSpin G-25 Columns containing Sephadex G-25 DNA grade F resin (Cytiva or GE Healthcare). In order to fold G4 and duplex structures, the labeled displaced strand was mixed with 1.5 times excess unlabeled loading strand in a buffer containing 100 mM KCl or LiCl. The mixture was then heated at 95 °C for 6 min and slowly cooled to room temperature to promote the formation of a duplex as well as a G4 structure. Full length genotype con1b NS3 was purified as explained in (38), except only the first and the second talon metal affinity columns were used. The purified protein was stored in a buffer containing 25 mM Hepes, 100 mM NaCl, 1 mM EDTA, 5 mM β-mercaptoethanol, and 20% glycerol. The protein concentration was calculated by UV absorbance at 280 nm with extinction coefficient value of 64,000 M^−1^ cm^−1^.

### CD spectroscopy

CD spectroscopy was conducted to confirm the ability of the guanine-rich RNA sequences (Table S1) to fold into G4 structures (34). CD spectra were generated as described in references (40, 57, 58) with minor modifications. Here, 9 μM or 10.2 μM guanine-rich RNA oligonucleotides were suspended in a G4-folding buffer containing 10 mM Hepes, pH 7.5, 1 mM EDTA, pH 8.0, and 100 mM KCl, then heated at 95 °C for 6 min and slowly cooled to room temperature before using a Jasco J-715 spectropolarimeter (JASCO) to measure the CD spectra. The scanning speed of the spectropolarimeter was set at 100 nm/min for a wavelength range of 200 to 320 nm.

### Fluorescence anisotropy–binding assay

Binding assays were conducted as described (59). In short, all of the Fluorescein Amidite–labeled G-rich RNA substrates (Table S1) were suspended in a G4-folding buffer containing 100 mM KCl, heated at 95 °C for 6 min and slowly cooled to room temperature. The folded G4 substrates (2 nM final concentration, unless stated otherwise) were preincubated in binding buffer (25 mM MOPS, pH 6.5; 100 mM KCl, 0.1 mM EDTA, 2 mM β-mercaptoethanol, 0.1 mg/ml bovine serum albumin in DEPC-treated water) with increasing concentrations of NS3 in a 96-well plate for 30 min at 37 °C. A PerkinElmer 1420 multilabel counter victor ^3^V plate reader was used to determine the polarization of NS3-G4 complex with 485 nm and 535 nm excitation and emission wavelengths, respectively. The polarization measurements were converted to fluorescence anisotropy values, which were then fit to a quadratic equation (unless otherwise stated) to acquire dissociation constants (*K*_*d*_).

### Excess enzyme-catalyzed dsRNA and G4 reporter duplex unwinding assay

The concentrations mentioned for the excess enzyme-catalyzed dsRNA and G4RNA reporter duplex unwinding assay are final. The dsRNA or a G4 reporter substrate (2 nM) in 100 mM KCl or LiCl was preincubated with 600 nM NS3 (unless otherwise specified) for 30 min at 37 °C in a reaction buffer containing 25 mM MOPS, pH 6.5, 100 mM KCl or LiCl, 0.1 mM EDTA, 2 mM β-mercaptoethanol, and 0.1 mg/ml bovine serum albumin. Simultaneously, in another reaction tube, 5 mM ATP, 10 mM MgCl_2_, and 60 nM trap substrate (Table S1) were preincubated together. For a single cycle reaction, the second reaction tube also contained 0.5 mM T_50_ in nucleotides as a protein trap. The reaction commenced when the contents of the two reaction tubes were combined. The reaction was terminated at increasing time points by addition of quench (100 mM EDTA and 1% SDS). For a time zero (blank) sample, equal volumes of samples from the NS3-substrate and ATP-Mg^+2^ tubes were taken out separately and then quenched into the same quench buffer immediately before the reaction was initiated (unless otherwise stated). For a heat control sample, half of the quenched zero-point sample was heated at 95 °C for 6 min in a separate vial and cooled down on ice. The products and the reactants of each of the samples (time-zero, heat control, and reaction samples) were separated by 20% native PAGE. A Typhoon Trio phosphorimager was used to image the samples on the gel, which was then quantified by ImageQuant software (GE Healthcare) for data analysis. Each datum point was normalized to the product measured at zero-time point. Data was fit to a single or a double exponential equation (unless otherwise stated) using KaleidaGraph software (Synergy Software).

### Enzyme-coupled spectrophotometric ATPase assay

An ATPase assay was performed as communicated in reference (59). The activity of NS3 was examined in an ATPase assay reaction buffer containing 50 mM MOPS, pH 6.5, 50 or 100 mM KCl, 10 mM MgCl_2_, 5 mM ATP, 4 mM phosphoenolpyruvate, 0.7 mg/ml NADH, and 10 u/ml pyruvate kinase and lactate dehydrogenase. Poly (U) (5 μM, 100 μM, or 250 μM) was added into the reaction to initiate hydrolysis of ATP by NS3. PK/LDH links the hydrolysis of ATP with the oxidation of NADH. The change in the concentration of NADH was determined by measuring the change in the UV absorbance of NADH at 380 nm. The rate of ATP hydrolysis is equivalent to the rate of change in the concentration of NADH. ATPase activity was calculated by dividing the rate of ATP hydrolysis by the amount of NS3 used in the reaction (50 nM) and is expressed in inverse of a second (s^−1^).

### G4RNA unfolding by trapping

The concentrations mentioned for G4RNA-unfolding trap assay are final. All substrates were mixed in a buffer containing 100 mM KCl, heated at 95 °C for 6 min, and slowly cooled to room temperature before using it in the unfolding reaction. In one reaction tube, 25 nM Fluorescein Amidite–labeled HCVG4A20, NEGG4A20, or NONHCVG4A20 substrate was incubated with 800 nM NS3 at 37 °C for 30 min in a reaction buffer containing 25 mM MOPS, pH 6.5, 100 mM KCl, 0.1 mM EDTA, 2 mM β-mercaptoethanol, and 0.1 mg/ml bovine serum albumin. In a second reaction tube, 5 mM ATP, 10 mM MgCl_2_, 750 nM Q-trap (complementary to the G-rich substrate) were incubated together at 37 °C for 30 min. The G4RNA-unfolding reaction was triggered by combining equal volumes of the contents of the two reaction tubes. The reaction was quenched at increasing time points by 100 mM EDTA, 1% SDS, and 60 μM C-trap (it sequesters excess Q trap). The zero-time point (blank) sample was collected immediately before the reaction was initiated. The partial duplex product was resolved from the G4RNA substrate with 20% native PAGE. The gel was then visualized using a Typhoon Trio phosphorimaging system and quantified using ImageQuant software. The fraction product formed was determined using the formula mentioned in reference (58) for the G4DNA-unfolding trap assay.

## Data availability

All data are contained within the article.

## Supporting information

This article contains supporting information.

## Conflict of interest

The authors declare that they have no conflicts of interest with the contents of this article.

## Supporting information

Figure S1

Figure s2

## References

[1] Manns M.P., Buti M., Gane E., Pawlotsky J.M., Razavi H., Terrault N. (2017). Hepatitis C virus infection. Nat. Rev. Dis. Prim..

[2] Polaris Observatory HCV Collaborators (2017). Global prevalence and genotype distribution of hepatitis C virus infection in 2015: a modelling study. Lancet Gastroenterol. Hepatol..

[3] Paul D., Madan V., Bartenschlager R. (2014). Hepatitis C virus RNA replication and assembly: living on the fat of the land. Cell Host Microbe.

[4] Kolykhalov A.A., Mihalik K., Feinstone S.M., Rice C.M. (2000). Hepatitis C virus-encoded enzymatic activities and conserved RNA elements in the 3’ nontranslated region are essential for virus replication *in vivo*. J. Virol..

[5] Scheel T.K.H., Rice C.M. (2013). Understanding the hepatitis C virus life cycle paves the way for highly effective therapies. Nat. Med..

[6] Raney K.D., Sharma S.D., Moustafa I.M., Cameron C.E. (2010). Hepatitis C virus non-structural protein 3 (HCV NS3): a multifunctional antiviral target. J. Biol. Chem..

[7] Byrd A.K., Raney K.D. (2012). Superfamily 2 helicases. Front. Biosci..

[8] Frick D.N. (2007). The hepatitis C virus NS3 protein: a model RNA helicase and potential drug target. Curr. Issues Mol. Biol..

[9] Mendoza O., Bourdoncle A., Bouí J.-B., Brosh R.M., Mergny J.-L. (2016). Survey and summary G-quadruplexes and helicases. Nucl. Acids Res..

[10] Rhodes D., Lipps H.J. (2015). G-quadruplexes and their regulatory roles in Biology. Nucl. Acids Res..

[11] Kharel P., Balaratnam S., Beals N., Basu S. (2020). The role of RNA G-quadruplexes in human diseases and therapeutic strategies. Wiley Inter. Rev. RNA..

[12] Song J., Perreault J.-P., Topisirovic I., Ephane Richard S. (2016). RNA G-quadruplexes and their potential regulatory roles in translation. Translation.

[13] Varshney D., Spiegel J., Zyner K., Tannahill D., Balasubramanian S. (2020). The regulation and functions of DNA and RNA G-quadruplexes. Nat. Rev. Mol. Cell Biol..

[14] Lavezzo E., Berselli M., Frasson I., Perrone R., Palù G., Brazzale A.R. (2018). G-Quadruplex forming sequences in the genome of all known human viruses: a comprehensive guide. PLoS Comput. Biol..

[15] Wang S.R., Min Y.Q., Wang J.Q., Liu C.X., Fu B.S., Wu F. (2016). A highly conserved G-rich consensus sequence in hepatitis C virus core gene represents a new anti–hepatitis C target. Sci. Adv..

[16] Jaubert C., Bedrat A., Bartolucci L., Di Primo C., Ventura M., Mergny J.L. (2018). RNA synthesis is modulated by G-quadruplex formation in Hepatitis C virus negative RNA strand. Sci. Rep..

[17] Artusi S., Nadai M., Perrone R., Biasolo M.A., Palù G., Flamand L. (2015). The Herpes Simplex Virus-1 genome contains multiple clusters of repeated G-quadruplex: implications for the antiviral activity of a G-quadruplex ligand. Antivir. Res..

[18] Madireddy A., Purushothaman P., Loosbroock C.P., Robertson E.S., Schildkraut C.L., Verma S.C. (2016). G-quadruplex-interacting compounds alter latent DNA replication and episomal persistence of KSHV. Nucl. Acids Res..

[19] Perrone R. (2013). A dynamic G-quadruplex region regulates the HIV-1 long terminal repeat promoter. J. Med. Chem..

[20] Sauer M., Paeschke K. (2017). G-quadruplex unwinding helicases and their function *in vivo*. Biochem. Soc. Trans..

[21] Caterino M., Paeschke K. (2022). Action and function of helicases on RNA G-quadruplexes. Methods.

[22] Booy E.P., Meier M., Okun N., Novakowski S.K., Xiong S., Stetefeld J. (2012). The RNA helicase RHAU (DHX36) unwinds a G4-quadruplex in human telomerase RNA and promotes the formation of the P1 helix template boundary. Nucl. Acids Res..

[23] Chakraborty P., Grosse F. (2011). Human DHX9 helicase preferentially unwinds RNA-containing displacement loops (R-loops) and G-quadruplexes. DNA Repair (Amst).

[24] Creacy S.D., Routh E.D., Iwamoto F., Nagamine Y., Akman S.A., Vaughn J.P. (2008). G4 resolvase 1 binds both DNA and RNA tetramolecular quadruplex with high affinity and is the major source of tetramolecular quadruplex G4-DNA and G4-RNA resolving activity in HeLa cell lysates. J. Biol. Chem..

[25] Tippana R., Chen M.C., Demeshkina N.A., Ferré-D’Amaré A.R., Myong S. (2019). RNA G-quadruplex is resolved by repetitive and ATP-dependent mechanism of DHX36. Nat. Commun..

[26] Liu H., Lu Y.N., Paul T., Periz G., Banco M.T., Ferré-D’Amaré A.R. (2021). A helicase unwinds hexanucleotide repeat RNA G-quadruplexes and facilitates repeat-associated non-AUG translation. J. Am. Chem. Soc..

[27] Huang W., Smaldino P.J., Zhang Q., Miller L.D., Cao P., Stadelman K. (2012). Yin Yang 1 contains G-quadruplex structures in its promoter and 5′-UTR and its expression is modulated by G4 resolvase 1. Nucl. Acids Res..

[28] Gosert R., Egger D., Lohmann V., Bartenschlager R., Blum H.E., Bienz K. (2003). Identification of the hepatitis C virus RNA replication complex in Huh-7 cells harboring subgenomic replicons. J. Virol..

[29] Quinkert D., Bartenschlager R., Lohmann V. (2005). Quantitative analysis of the hepatitis C virus replication complex. J. Virol..

[30] Pandey S., Agarwala P., Maiti S. (2013). Effect of loops and G-quartets on the stability of RNA G-quadruplexes. J. Phys. Chem. B..

[31] Gwack Y., Kim D.W., Han J.H., Choe J. (1996). Characterization of RNA binding activity and RNA helicase activity of the hepatitis C virus NS3 protein. Biochem. Biophys. Res. Commun..

[32] Tai C.L., Chi W.K., Chen D.S., Hwang L.H. (1996). The helicase activity associated with hepatitis C virus nonstructural protein 3 (NS3). J. Virol..

[33] Levin M.K., Gurjar M., Patel S.S. (2005). A Brownian motor mechanism of translocation and strand separation by hepatitis C virus helicase. Nat. Struct. Mol. Biol..

[34] Kypr J., Kejnovská I., Renčiuk D., Vorlíčková M. (2009). Circular dichroism and conformational polymorphism of DNA. Nucl. Acids Res..

[35] Oliver A.W., Kneale G.G. (1999). Structural characterization of DNA and RNA sequences recognized by the gene 5 protein of bacteriophage fd. Biochem. J..

[36] Del Villar-Guerra R., Trent J.O., Chaires J.B. (2018). G-quadruplex secondary structure obtained from circular dichroism spectroscopy. Angew. Chem. Int. Ed. Engl..

[37] Beran R.K.F., Serebrov V., Pyle A.M. (2007). The serine protease domain of hepatitis C viral NS3 activates RNA helicase activity by promoting the binding of RNA substrate. J. Biol. Chem..

[38] Raney V.M., Reynolds K.A., Harrison M.K., Harrison D.K., Cameron C.E., Raney K.D. (2012). Binding by the hepatitis C virus NS3 helicase partially melts duplex DNA. Biochemistry.

[39] Levin M.K., Patel S.S. (2002). Helicase from hepatitis C virus, energetics of DNA binding. J. Biol. Chem..

[40] Griffin W.C., Gao J., Byrd A.K., Chib S., Raney K.D. (2017). A biochemical and biophysical model of G-quadruplex DNA recognition by positive coactivator of transcription 4. J. Biol. Chem..

[41] Yangyuoru P.M., Bradburn D.A., Liu Z., Xiao T.S., Russell R. (2018). The G-quadruplex (G4) resolvase DHX36 efficiently and specifically disrupts DNA G4s *via* a translocation-based helicase mechanism. J. Biol. Chem..

[42] Reynolds K.A., Cameron C.E., Raney K.D. (2015). Melting of duplex DNA in the absence of ATP by NS3 helicase domain through specific interaction with a single-strand/double-strand junction. Biochemistry.

[43] Bhattacharyya D., Arachchilage G.M., Basu S. (2016). Metal cations in G-quadruplex folding and stability. Front. Chem..

[44] Zhou T., Ren X., Adams R.L., Pyle A.M. (2018). NS3 from hepatitis C virus strain JFH-1 is an unusually robust helicase that is primed to bind and unwind viral RNA. J. Virol..

[45] Gao J., Byrd A.K., Zybailov B.L., Marecki J.C., Guderyon M.J., Edwards A.D. (2019). DEAD-box RNA helicases Dbp2, Ded1 and Mss116 bind to G-quadruplex nucleic acids and destabilize G-quadruplex RNA. Chem. Commun..

[46] Rajagopal V., Patel S.S. (2008). Single strand binding proteins increase the processivity of DNA unwinding by the hepatitis C virus helicase. J. Mol. Biol..

[47] Zhang C., Cai Z., Kim Y.-C., Kumar R., Yuan F., Shi P.-Y. (2005). Stimulation of hepatitis C virus (HCV) nonstructural protein 3 (NS3) helicase activity by the NS3 protease domain and by HCV RNA-dependent RNA polymerase. J. Virol..

[48] Levin M.K., Wang Y.H., Patel S.S. (2004). The functional interaction of the hepatitis C virus helicase molecules is responsible for unwinding processivity. J. Biol. Chem..

[49] Byrd A.K., Raney K.D. (2005). Increasing the length of the single-stranded overhang enhances unwinding of duplex DNA by bacteriophage T4 Dda helicase. Biochemistry.

[50] Ramanagoudr-Bhojappa R., Chib S., Byrd A.K., Aarattuthodiyil S., Pandey M., Patel S.S. (2013). Yeast Pif1 helicase exhibits a one-base-pair stepping mechanism for unwinding duplex DNA. J. Biol. Chem..

[51] Cui H., Zhang L. (2020). G-quadruplexes are present in human coronaviruses including SARS-CoV-2. Front. Microbiol..

[52] Fleming A.M., Ding Y., Alenko A., Burrows C.J. (2016). Zika virus genomic RNA possesses conserved G-quadruplexes characteristic of the flaviviridae family. ACS Infect. Dis..

[53] Murat P., Zhong J., Lekieffre L., Cowieson N.P., Clancy J.L., Preiss T. (2014). G-quadruplexes regulate Epstein-Barr virus-encoded nuclear antigen 1 mRNA translation. Nat. Chem. Biol..

[54] Perrone R., Nadai M., Poe J.A., Frasson I., Palumbo M., Palù G. (2013). formation of a unique cluster of G-quadruplex structures in the HIV-1 nef coding region: implications for antiviral activity. PLoS One.

[55] Marecki J.C., Belachew B., Gao J., Raney K.D. (2021). RNA helicases required for viral propagation in humans. Enzymes.

[56] Byrd A.K., Raney K.D. (2004). Protein displacement by an assembly of helicase molecules aligned along single-stranded DNA. Nat. Struct. Mol. Biol..

[57] Byrd A.K., Bell M.R., Raney K.D. (2018). Pif1 helicase unfolding of G-quadruplex DNA is highly dependent on sequence and reaction conditions. J. Biol. Chem..

[58] Edwards A.D., Marecki J.C., Byrd A.K., Gao J., Raney K.D. (2021). G-Quadruplex loops regulate PARP-1 enzymatic activation. Nucl. Acids Res..

[59] Byrd A.K., Raney K.D. (2015). A parallel quadruplex DNA is bound tightly but unfolded slowly by Pif1 helicase. J. Biol. Chem..

