# Supplementary material for: Hepatitis C virus nonstructural protein NS3 unfolds viral G-quadruplex RNA structures
Source: J Biol Chem. 2022 Sep 13;298(11):102486. doi: 10.1016/j.jbc.2022.102486 (PMC9582721; doi:10.1016/j.jbc.2022.102486)
Supplement: Supporting information [file mmc1.docx]

**Hepatitis C virus non-structural protein NS3 unfolds viral G-quadruplex RNA structures**

**Binyam Belachew, Jun Gao, Alicia K. Byrd, and Kevin D. Raney**^*^

From the Department of Biochemistry and Molecular Biology, University of Arkansas for Medical Sciences, Little Rock, Arkansas 72205

^*^Author to whom correspondence should be addressed. Tel.: 501-686-5244; Fax: 501-686-8169; E-mail: raneykevind@uams.edu

**Supporting information**

**Figure S1: NS3 binds tightly to A_20_ oligonucleotide (supporting information for Figure 2).** The fluorescence anisotropy measurements of NS3 with A_20_ are shown above from Figure 2. These measurements are fit to a two-sites binding model. *K_d_*_1_= 4.9 with 95% CI [3.4, 6.0] and K_d2_ = 860.3 with 95% CI [30.2, 14827756310003].

**Figure S2: A G4 reporter duplex substrate preparation (supporting information for Figure 4):** A G4 reporter duplex substrate was made by mixing an unlabeled loading strand that consisted of a duplex forming region, G-rich sequences, and A_20_ 3'overhang with a radiolabeled displaced strand (* indicates labeling) in 100 mM KCl. The G4 reporter substrate contained a G4 structure flanked by a duplex region and A_20_ 3' overhang.

**Figure S3: Defining the enzyme concentration that saturates the G4 reporter duplex unwinding reaction (supporting information for Figure 4).** A G4 reporter duplex unwinding reaction was conducted with radiolabeled HCVG4-A_20_ reporter duplex substrate (2 nM) and varying concentrations of NS3. The above gel image shows the products formed at increasing time points from 400, 600, 800, and 1000 nM NS3. The polyacrylamide gel electrophoresis was conducted for 3 hrs. at 22 mA. The time zero (blank) samples were prepared before the 30 min. pre-incubation.

**Figure S4:** **Pyridostatin (PDS) has significantly higher effect on NS3 catalyzed HCVG4-A_20_ reporter duplex unwinding than on MUTHCVG4-A_20_ (supporting information for Figure 5).** The data for NS3 unwinding of MUTHCVG4-A_20_ under standard conditions described in Figure 5 (A; green circles) or in the presence of 0.25 µM PDS (A; red circles) or in the absence of ATP (A; blue circles) is shown in (A). The amount of unwound MUTHCVG4-A_20_ or HCVG4-A_20_ in the presence of PDS was presented in (B) normalized to the amount unwound in the absence of PDS. The experiments were performed in triplicate. Error bars signify standard deviation. The difference between two groups is statically significant when P ≤ 0.001 (***). P values were calculated from independent-samples t-tests.

**Figure S5:** **The HCV G-rich sequence within HCVG4-A_20_ reporter duplex are responsible for reducing the observed unfolding activity of NS3 in 100 mM KCl (supporting information for Figure 5).** The G-rich sequence within HCVG4-A_20_ reporter duplex was deleted to form a substrate with 25-mer duplex and 20-mer 3' overhang (DELHCVG4-A_20_). The product formed from HCVG4-A_20_ (green circles) or DELHCVG4-A_20_ (black circles) unwinding by NS3 is presented in line graph. The data for HCVG4-A_20_ are replotted from Fig. 5 for comparison. The experiments were conducted in triplicate, and error bars depict standard deviation.

**Figure S6: Intermolecular G4 formation by HCVG4-A_20_, NEGG4-A_20_ and NONHCVG4-A_20_ reporter duplex substrates in a buffer containing 100 mM KCl or 100 mM LiCl (supporting information for Figure 9).** The products formed at increasing time points from a radiolabeled HCVG4-A_20_ (A), NEGG4-A_20_ (C) or NONHCVG4-A_20_ (E) reporter duplex unwinding reaction in 100 mM KCl buffer are shown on a 20 % polyacrylamide gel. Orange arrows (A, C, & E) point at substrates of higher order structures (intermolecular G4 structures). The gel images in panel B, D & F show the products formed from HCVG4-A_20_, NEGG4-A_20_ and NONHCVG4-A_20_ reporter duplex unwinding reactions conducted in a buffer containing 100 mM LiCl, respectively. The gel images for + 600 nM NS3 and –NS3 in panel (A) are reused from Fig. 5B for comparison. In addition, the gel image in panel (B) is re-represented from Fig. 6C for comparison.

**Figure S7: Determining the saturating enzyme concentration for the substrate used in G4RNA trapping reaction (supporting information for Figure 10).** Panels (A & B) show the products obtained from a G4RNA trapping reaction in the presence of 600 nM (blue circles (A) and red squares (B)), 800 nM (black circles (A) and grey circles (B)), or 1000 nM (red circles (A and B)) NS3 and 25 nM FAM labeled HCVG4-A_20_. The amount of products formed from 800 nM and 1000 nM NS3 are not significantly different (B). The experiments were performed at least in triplicate. Error bars signify standard deviation. P value > 0.05 indicates the difference between two groups is not statistically significant (ns). P values were calculated from independent-samples t-tests.
