# Supplementary figures and images for: Hepatitis C virus nonstructural protein NS3 unfolds viral G-quadruplex RNA structures
Source: J Biol Chem. 2022 Sep 13;298(11):102486. doi: 10.1016/j.jbc.2022.102486 (PMC9582721; doi:10.1016/j.jbc.2022.102486)

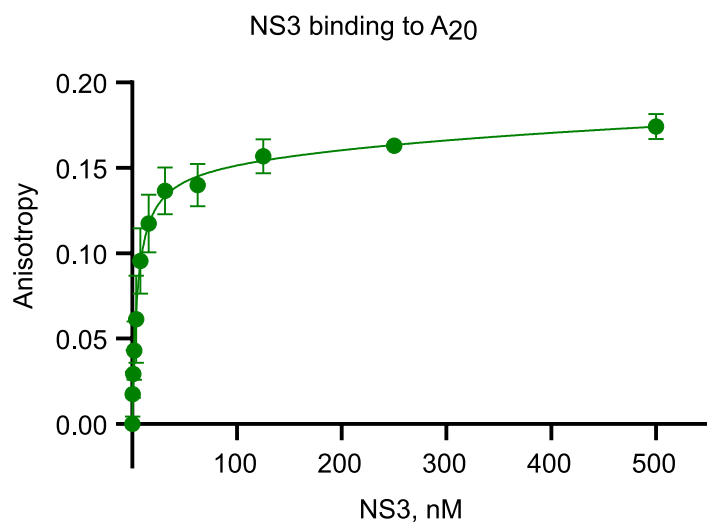

Supplement: Figure S1 [file mmc3.pdf]

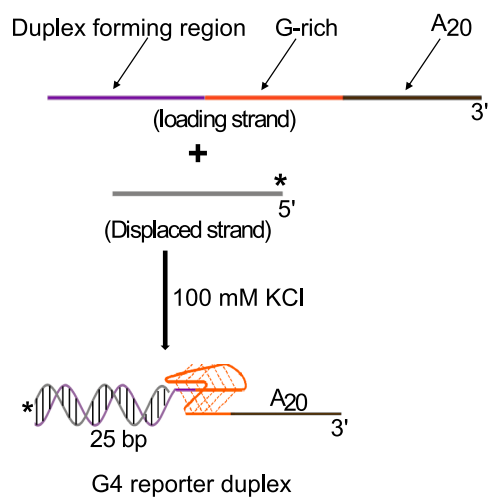

Supplement: Figure s2 [file mmc4.pdf]

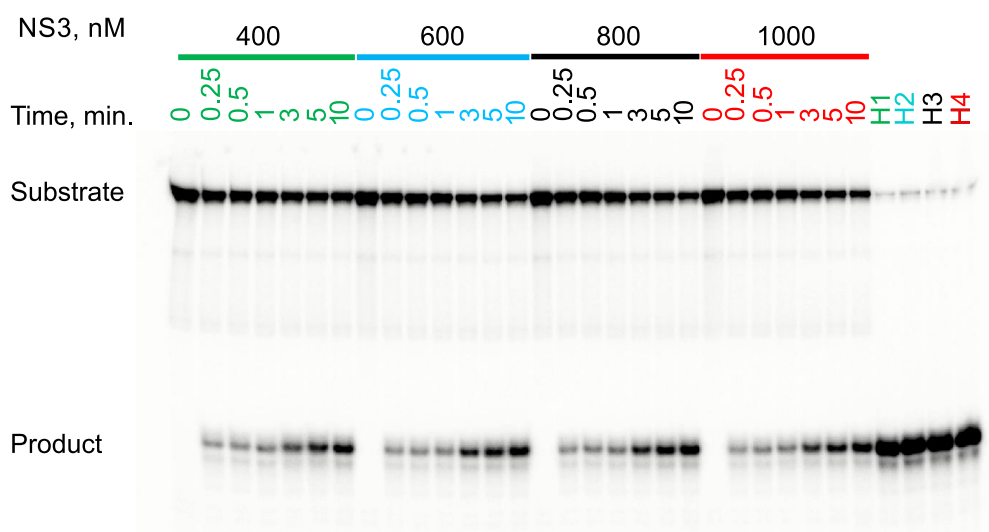

Supplement: Figure S3 [file mmc5.pdf]

**A** MUTHCVG4-A<sub>20</sub> reporter duplex

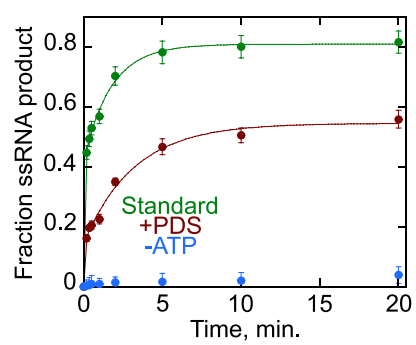

**B**

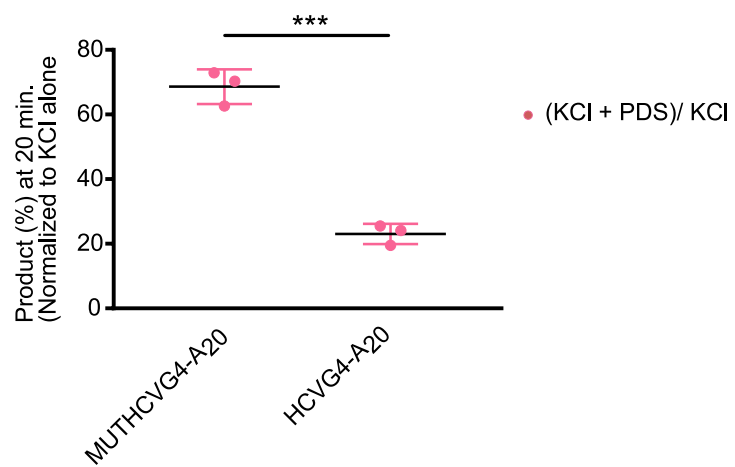

Supplement: Figure S4 [file mmc6.pdf]

A G4 reporter duplex and a control duplex unwinding

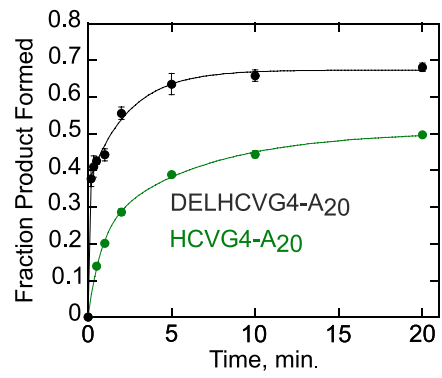

Supplement: Figure S5 [file mmc7.pdf]

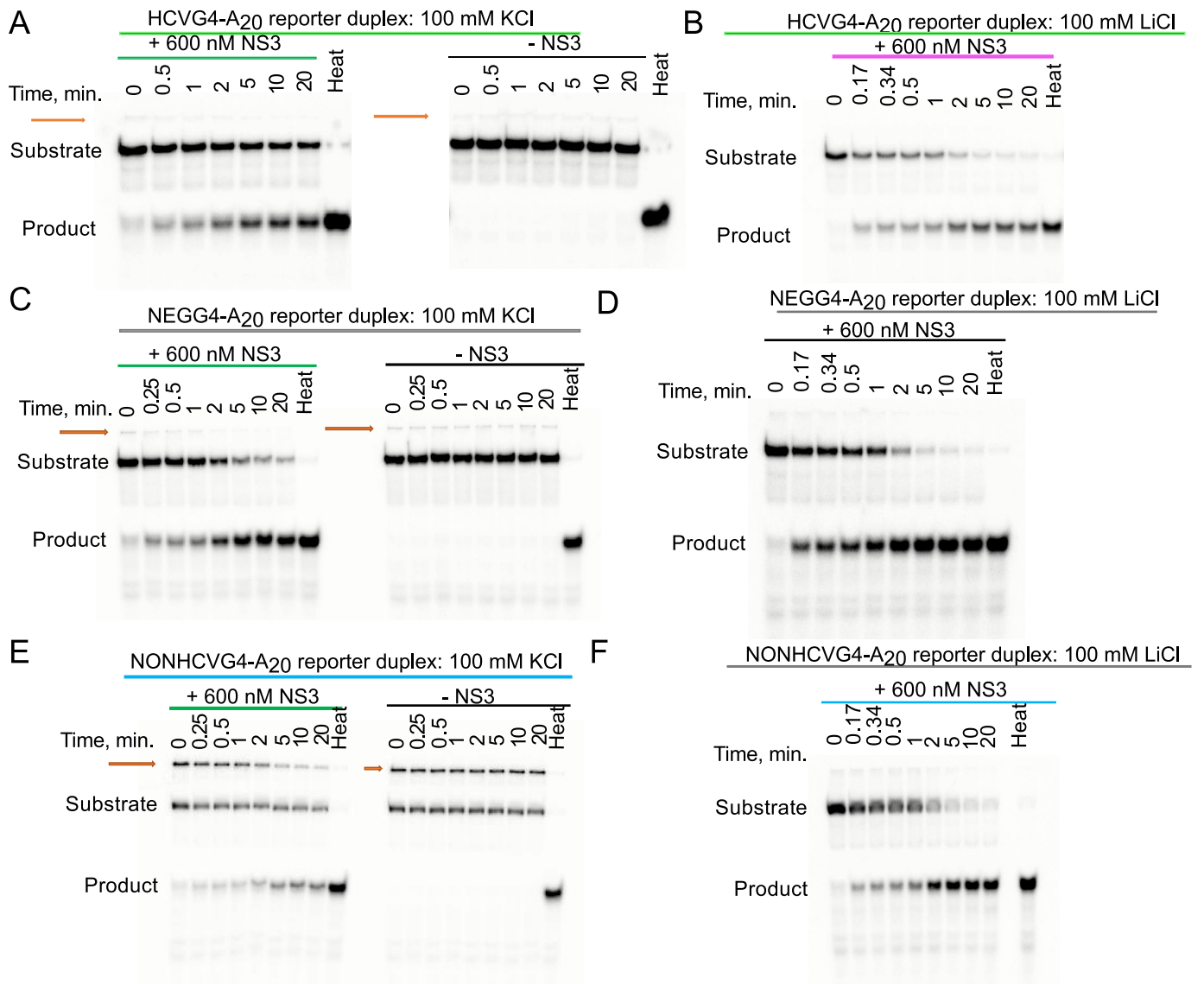

Supplement: Figure S6 [file mmc8.pdf]

A

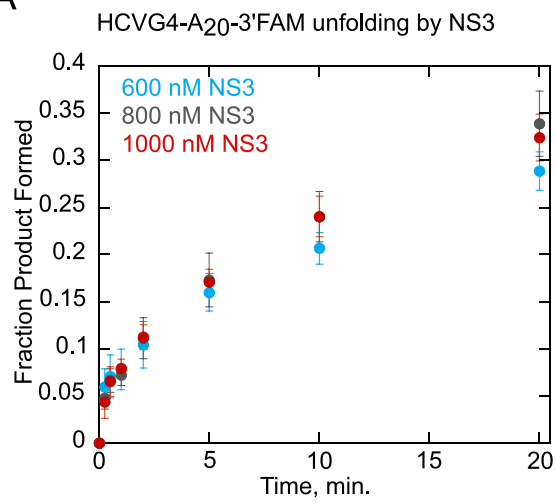

B

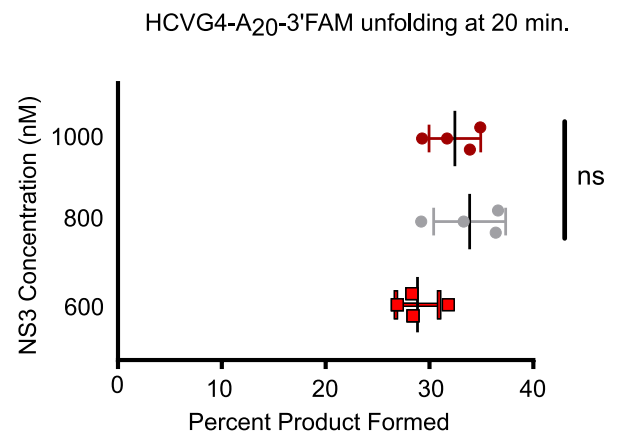

Supplement: Figure S7 [file mmc9.pdf]
